# Integrating the Consolidated Framework for Implementation Research (CFIR) into a Culturally Responsive Evaluation (CRE) Approach to Conduct Mixed-Method Evaluations of Diabetes Prevention and Management Programs Reaching Underresourced Populations and Communities

**DOI:** 10.1007/s11121-023-01509-1

**Published:** 2023-03-22

**Authors:** Sara R. Jacobs, LaShawn Glasgow, Peter Amico, Kimberly D. Farris, Gia Rutledge, Bryce D. Smith

**Affiliations:** 1 RTI International, Research Triangle Park, NC USA; 2 Amico Consulting, Orlando, FL USA; 3 Centers for Disease Control and Prevention, Atlanta, GA USA

**Keywords:** Culturally responsive evaluation, Implementation science, Mixed-methods evaluations, Diabetes prevention and management

## Abstract

Diabetes is a significant population health threat. Evidence-based interventions, such as the Centers for Disease Control and Prevention’s National Diabetes Prevention Program and diabetes self-management education and support programs, can help prevent, delay, or manage the disease. However, participation is suboptimal, especially among populations who are at an increased risk of developing diabetes. Evaluations of programs reaching populations who are medically underserved or people with lower incomes can help elucidate how best to tailor evidence-based interventions, but it is also important for evaluations to account for cultural and contextual factors. Culturally responsive evaluation (CRE) is a framework for centering an evaluation in the culture of the programs being evaluated. We integrated CRE with implementation and outcome constructs from the Adapted Consolidated Framework for Implementation Research (CFIR) to ensure that the evaluation produced useful evidence for putting evidence-based diabetes interventions to use in real-world settings, reaching populations who are at an increased risk of developing diabetes. The paper provides an overview of how we integrated CRE and CFIR approaches to conduct mixed-methods evaluations of evidence-based diabetes interventions.

## Introduction

More than 37 million U.S. adults have diabetes, and they face medical expenditures more than double those of individuals without diabetes (Centers for Disease Control and Prevention (CDC), 2022). Type 2 diabetes disproportionately impacts racial and ethnic minorities (CDC, 2017). For example, the incidence of type 2 diabetes among Black persons (12.6%) and Hispanic or Latino persons (11.8%) is higher than that of non-Hispanic White persons (7.1%). In addition, non-Hispanic Black and non-Hispanic White adults are also more likely to experience morbidity, such as retinopathy from the disease; in addition, non-Hispanic Black persons are 2.3 times more likely to be hospitalized for lower limb amputations as compared to non-Hispanic Whites (CDC, 2018, 2021; Spanakis & Golden, 2013).

Evidence has demonstrated that lifestyle change interventions are effective in reducing the risk of developing type 2 diabetes among persons with prediabetes and to help manage diabetes among persons already diagnosed with diabetes (Knowler et al., 2002). For example, the CDC established the National Diabetes Prevention Program (National DPP), which is a year-long intervention to assist persons with prediabetes to make lifestyle changes associated with preventing or delaying the onset of type 2 diabetes (Albright & Gregg, 2013). Specifically, the National DPP has multiple components, including lifestyle coach training, a quality control program (the Diabetes Prevention Recognition Program), workforce development, a health marketing campaign, and an initiative to develop intervention sites to deliver the lifestyle change program.

Diabetes self-management education and support (DSMES) programs help people manage diabetes, prevent complications, and, in turn, help lower the health care costs of diabetes (Powers et al., 2015). The American Diabetes Association (ADA) and Association of Diabetes Care & Education Specialists (ADCES) (formerly known as the American Association of Diabetes Educators) define DSMES as the “process of facilitating the knowledge, skill, or ability necessary for diabetes self-care” (Powers et al., 2017). Support in DSMES refers to any type of behavioral, clinical, educational, or psychosocial services provided to help persons with diabetes implement and maintain self-management behaviors, including healthy eating, taking medication, being active, and healthy coping (Powers et al., 2017).

Despite the proven effectiveness of lifestyle change and DSMES programs across racial and ethnic groups, enrollment in these interventions among racial and ethnic minority populations is lower than among non-Hispanic White persons. For example, from 2012 to 2019, only 13.2% of total enrollment into the National DPP self-identified as Hispanic or Latino compared with 86.8% who identified as non-Hispanic and 13.1% who self-identified as Black or African American compared with 64.6% who identified as White (Cannon et al., 2022). In addition, in 2015, only 54.4% of adults aged 18 years or older with diabetes reported having ever attended a DSMES class with lower utilization reported among uninsured adults with diabetes and Medicaid recipients (CDC, 2018; Powers et al., 2017).

There is an immediate need for more evidence on successful enrollment strategies for both the National DPP and DSMES programs. Therefore, the current study is a collaboration between CDC’s Division of Diabetes Translation (DDT) and RTI International to conduct rapid evaluations of National DPP lifestyle change and DSMES programs for individuals who are medically underserved or who live in underresourced areas. Rapid evaluation is an approach designed to quickly and systematically conduct an evaluation when time or resources are limited (McNall & Foster-Fishman, 2007). Rapid evaluations often include a participatory approach designed to evaluate the implementation of programs in a manner that accounts for cultural and contextual factors. The goal of this paper is to describe our rapid evaluation approach in which we integrated components of an adaptation of a well-used, implementation science–based framework, the Consolidated Framework for Implementation Science (CFIR) (Damschroder et al., 2009), with the culturally responsive evaluation (CRE) framework to evaluate two National DPP lifestyle change program providers and two DSMES programs. All four programs were in existence at least 5 years before we conducted the rapid evaluations. The rapid evaluations were designed to gather practice-based evidence to enhance the implementation of these programs in real-world settings.

## Culturally Responsive Evaluation

**Fig. 1:**
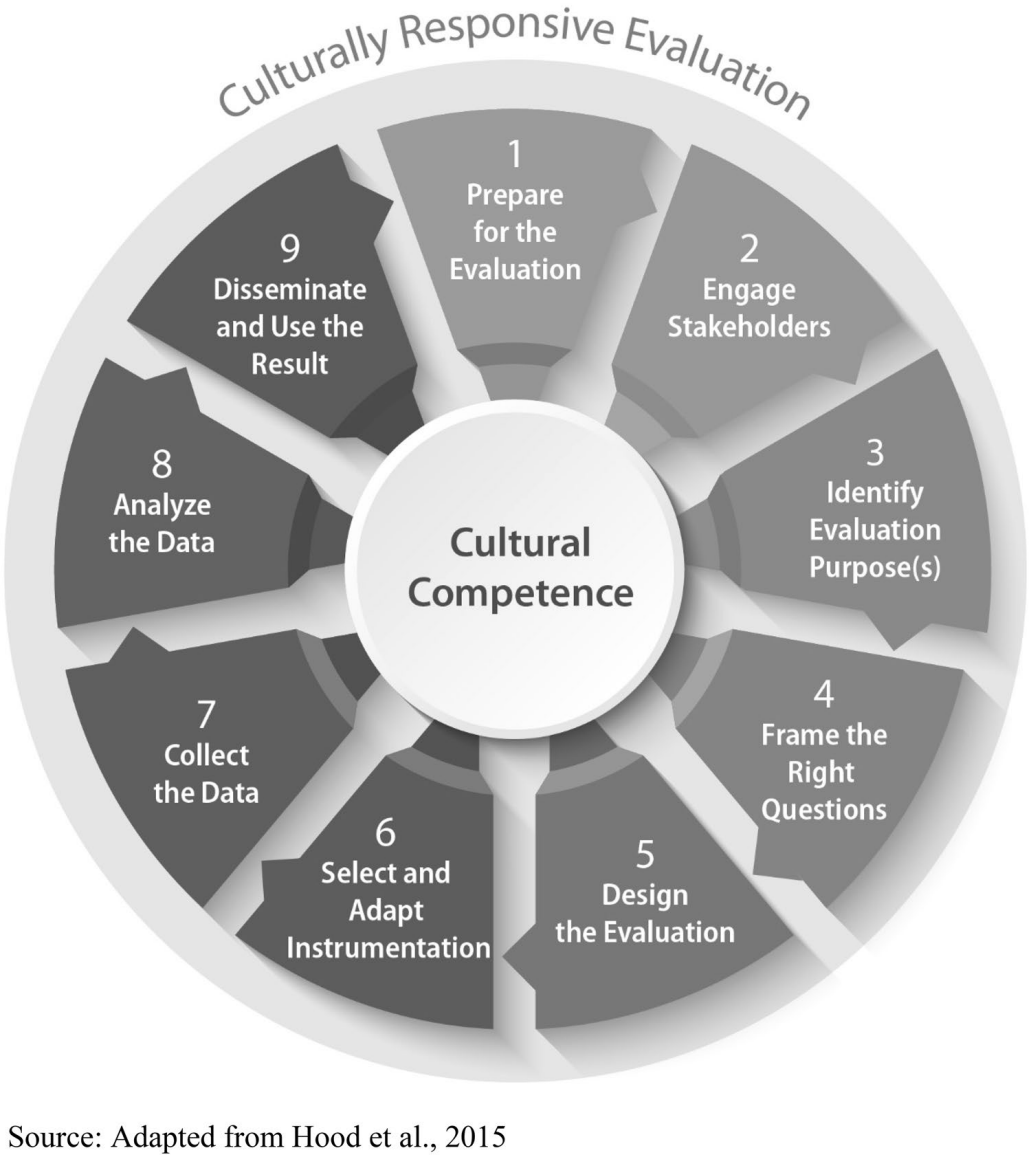
CRE framework

Given our goal was to conduct rapid evaluations of National DPP and DSMES programs located in medically underserved and underresourced communities, we based our approach on a CRE framework. CRE is a framework for centering an evaluation into the culture and community context in which it is taking place (Frierson et al., 2010). CRE recognizes that demographic, sociopolitical, and contextual dimensions, locations, perspectives, and characteristics of culture matter fundamentally in evaluation (Hood, 2001; Hopson, 2009). Additionally, CRE is designed to integrate equity in the evaluation process (Hood et al., 2015). CRE itself does not consist of a unique series of steps apart from other evaluation approaches; the distinction of CRE is in how each stage of the evaluation is operationalized (Hood et al., 2015). We followed the principles and nine stages of CRE as outlined by Frierson et al. (2002, 2010): preparing for the evaluation, engaging stakeholders, identifying evaluation purposes, framing the right questions, designing the evaluation, selecting and adapting instrumentation, collecting the data, analyzing the data, and disseminating and using the results. Figure 1 provides an overview of the nine steps involved in CRE.

## Consolidated Framework for Implementation Research

**Fig. 2:**
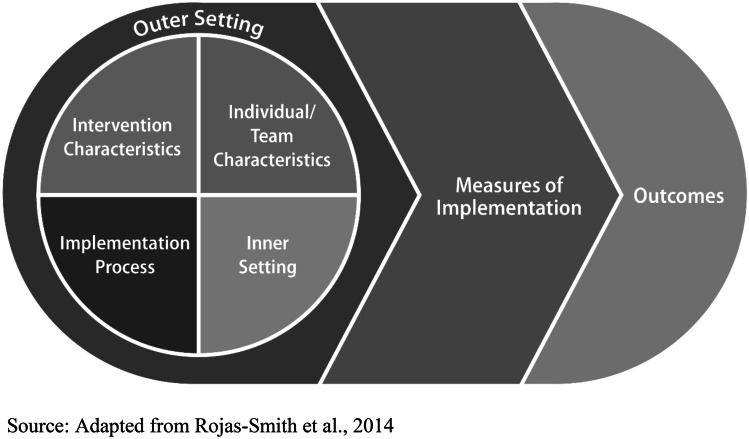
Adapted Consolidated Framework for Implementation Research (CFIR)

While CRE was used to guide the overall evaluation approach, we needed an additional framework to help capture facilitators that may be associated with successful implementation of the lifestyle change and DSMES programs in the four communities. Although there are more recent examples of implementation science frameworks and recommendations that incorporate health equity domains (Baumann & Cabassa, 2020; Shelton et al., 2021; Woodward et al., 2021), the planning and evaluations were conducted in 2017 and 2018 when the field was more nascent. As a result, we selected the Adapted CFIR framework to examine potentially relevant multilevel factors and outcomes (see Fig. 2). Importantly, our work aligns with the recently published Health Equity Implementation Framework (Woodward et al., 2021) and current recommendations for incorporating equity domains in implementation science frameworks and studies (Baumann & Cabassa, 2020; Shelton et al., 2021). Furthermore, our approach complements these more recent frameworks and recommendations by demonstrating the application of health equity principles to mixed-method evaluations of real-world community-clinical linkage interventions.

CFIR and the Adapted CFIR have been used to evaluate other community-based prevention interventions, including the National DPP (Aziz et al., 2015; Damschroder et al., 2017; Manca et al., 2014). CFIR consists of five domains: outer setting, inner setting, intervention characteristics, characteristics of individuals involved, and implementation process (Damschroder et al., 2009). The outer setting includes the economic, political, and social contexts of the communities, and the inner setting includes specific features of the organization itself, such as organizational climate, culture, and structure (Damschroder et al., 2009). Intervention characteristics include delivery mode and any support tools incorporated into the program, and characteristics of the program staff, including educators and lifestyle coaches, and of the intended participants are addressed under the individuals’ domain (Damschroder et al., 2009). The implementation process domain includes the strategies used to develop and deliver the intervention (Damschroder et al., 2009). The Adapted CFIR adds additional outcome constructs to the original framework (Rojas Smith et al., 2014). The added outcome constructs focus on the quality and consistency of program delivery and the scalability of the intervention, as well as enrollment in the program, retention, and costs.

By incorporating the Adapted CFIR into our CRE approach, we were able to quickly develop evaluation questions and data collection instruments that both addressed key cultural considerations and ensured we collected evidence that could help us translate the findings into practice-based guides to support the implementation of the National DPP lifestyle change and DSMES programs in real-world settings.

## Integrating CRE and CFIR

In this section, we walk through the nine steps associated with conducting a rapid CRE, specifically focusing on where and how we integrated components of the Adapted CFIR into our approach. We incorporated the Adapted CFIR into three CRE steps: framing research questions, developing and adapting data collection instruments, and analyzing the data.

### Step 1: Prepare for the Evaluation

CRE principles for evaluation planning include exploring the stories of the community where the intervention is implemented, as well as the experiences of the people who live in the community (Hood et al., 2015). Before the rapid evaluations, sites previously participated in a systematic screening and assessment that was conducted by CDC and the National Opinion Research Center and included phone interviews and site visits. Key evaluability criteria included CDC recognition for National DPP lifestyle change program providers or ADA recognition/ADCES accreditation for DSMES programs, time in operation, populations of interest, enrollment, cultural tailoring, innovative strategies, evidence of effectiveness and sustainability, data system capacity, community partners, and organizational capacity (National Opinion Research Center, 2017). Ultimately, CDC selected the four organizations to participate in the rapid evaluations.

Prior to starting the actual rapid evaluations, we prepared for the evaluations by reviewing any materials the sites could share regarding their respective programs. Documents shared included progress reports, workplans, program materials, and the systematic screening and assessment site visit summary reports. These documents were helpful for the study team in preparing for the site visits by understanding the history of the organization, any details regarding the program, and the populations served by the programs.

### Step 2: Engage Stakeholders

CRE emphasizes the importance of building a climate of respect and trust with key stakeholders, including program staff (Frierson et al., 2010; Hood et al., 2015). For this study, directors of the lifestyle change and DSMES programs were the evaluation teams’ primary points of contact and were engaged during the duration of the rapid evaluation. We also executed written agreements with the programs that detailed the scope of work and data use terms to help establish trust with program staff. The agreements provided a summary of roles for both the evaluation team and program staff, including the collaborative development of the evaluation plans. Written agreements also identified site visit participants, described the data to be collected and provided to the evaluation team, and outlined the program staff’s role in providing feedback on the evaluation team’s analysis and written evaluation reports. Given the time commitment associated with participating in the evaluation, we provided stipends to the lifestyle change and DSMES programs for their time and participation.

### Step 3: Identify Evaluation Purpose(s)

Identifying the evaluation purpose includes considering how the purpose may benefit the program and community, as well as ensuring that the purpose is clearly understood by key stakeholders (Hood et al., 2015). The key purpose of the rapid evaluation, as stated by CDC, was to identify promising approaches for recruiting, enrolling, and retaining eligible individuals who are medically underserved or who live in underresourced areas in the lifestyle change and DSMES programs. This purpose drove our approach under subsequent evaluation steps. For example, interviews conducted under Step 6 included questions about how organizations had tailored their programs to recruit, enroll, and retain priority population groups. Then, promising practices gathered from the site visits were shared among the participating sites to help inform program improvements. Promising practices were also incorporated into practice-based guides that were developed for programs that work with populations and communities that are located in underresourced areas (Step 9).

### Step 4: Frame the Right Questions

CRE principles for developing the right evaluation questions include accounting for stakeholder priorities and asking questions that gather the perspectives of individuals involved in the program (Hood et al., 2015). The evaluation team met with local program directors during an initial phone call to gather information from sites on the specific evaluation questions that were relevant for their respective programs. For example, program directors and leaders were very interested in more detailed assessments of program outcomes that could be used to promote the value of their lifestyle change and DSMES programs within their communities. Integrating the Adapted CFIR into our CRE approach was critical for quickly developing evaluation questions that centered the evaluation in the local culture and context. Table 1 shows some of the CRE-framed evaluation questions organized by selected CFIR constructs.

**Table 1:** CRE-framed evaluation questions by selected CFIR constructs

**CFIR construct**	**CRE-framed evaluation question**
***Outer setting***	
Context	• How do contextual factors (e.g., geographic location, political and social climate, economic conditions) affect implementation of the programs?
Patient needs and resources	• To what extent are the programs aware of participant needs, particularly needs that are uniquely experienced by Black, African American, Latino, Hispanic, and African immigrant participants? • To what extent are the programs aware of barriers and facilitators to meeting participants’ needs?
External policies and incentives	• How have external policies, regulations, mandates, or recommendations affected the programs? Do these external policies affect population groups differently?
***Inner setting***	
Organizational culture	• How does organizational culture affect implementation?
Leadership engagement	• How does the commitment, involvement, and accountability of leaders and managers affect implementation?
Available resources	• What resources are necessary for program implementation, cultural tailoring, and maintenance?
Partnerships and networks	• How are the programs partnering with other external organizations to implement the programs?
***Individual/team characteristics***	
Self-efficacy	• To what extent do staff believe they have the capabilities, including cultural competency, required to implement the programs?
Other personal attributes	• What traits, skills, and competencies of coaches and educators are important for successful implementation?
***Intervention characteristics***	
Adaptability	• How are the programs adapted and tailored to meet the needs of Black, African American, Latino, Hispanic, and African immigrant participants?
Cost	• What costs are associated with implementing and maintaining the programs? • What proportion of costs are related to tailoring the recruitment and intervention strategies for Black, African American, Latino, Hispanic, and African immigrant participants?

We also included evaluation questions (Table 2) related to reach, dose, cost, and clinical outcomes, consistent with other adaptations of CFIR in the literature (). The outcome-related evaluation questions were not intended to prove the link between the lifestyle change and DSMES interventions and clinical outcomes as the lifestyle change and DSMES programs have an established evidence base (Albright & Gregg, 2013; Creamer et al., 2016; Powers et al., 2017; Ricci-Cabello et al., 2014). Rather, implementation and clinical outcome questions were included in the evaluation to confirm that the four programs were being implemented effectively and to demonstrate whether these programs that focused on people who were at a higher risk of developing diabetes or were at increased risk for diabetes complications were achieving the desired clinical outcomes.

**Table 2:** Implementation and clinical outcome evaluation questions

**Construct**	**CRE-framed evaluation question**
Reach	• What are the characteristics of the populations being served by the programs? • To what extent does enrollment vary by population group?
Dose	• What is the average number of sessions attended by participants? • What are the programs’ overall completion rates, and how do completion rates vary by population group and across program sites?
Clinical outcomes	• To what degree is participation in the programs associated with improved clinical outcomes (i.e., A1c, blood pressure, body mass index) among all participants and among different population groups?

### Step 5: Design the Evaluation

Mixed methods are recommended in CRE, which positions evaluators to gather the range of evidence that is valued by different stakeholders (Hood et al., 2015). The rapid evaluation design included both the collection of primary data and the analysis of secondary data. The evaluation team considered the rapid timeline, the availability of data, and the resources required to collect primary data. Primary data were collected during site visits. The site visits included semi-structured interviews, focus groups with a mix of participants from each site, and classroom observations. We also collected primary data regarding program startup and operations via a cost survey conducted during the site visit. In addition, we analyzed secondary data provided to us by the sites related to program participation and outcomes. To gather the perspectives of a broader range of stakeholders, we held two subject matter expert (SME) panels after the site visits—one for the National DPP lifestyle change programs and one for the DSMES programs. SMEs included experts who were implementing DSMES and National DPP programs in their communities, health equity researchers, evaluators, and representatives from CDC.

### Step 6: Select and Adapt Instrumentation

CRE recommends examining the validity of existing tools for different population groups, scrutinizing existing data collection instruments for cultural bias, and developing instruments if existing instruments are not culturally appropriate (Hood et al., 2015). Our evaluation team developed data collection instruments, and interview and focus group questions were designed to address key Adapted CFIR constructs (Table 3). As part of instrument development prior to data collection, the evaluation team engaged program directors at each of the sites to determine which topics might be most relevant for the different site visit participants. The evaluation team then constructed interview guides for each type of participant and focus group. Program directors then reviewed these guides to ensure use of culturally appropriate and relevant language for their specific communities. For example, one program director stressed the importance of referring to “Hispanic and Latino” versus “Hispanic/Latino” participants, as the distinction between Latinos’ Latin American descent and Hispanics’ descent from Spanish-speaking populations was important to acknowledge within their community (CDC, n.d.). Another site reported the importance of distinguishing what might be relevant for an African immigrant population versus African American or Black persons. In addition, we developed a cost survey to be administered at each of the four sites to collect data related to the startup and operating costs of implementing the program.

**Table 3:** Example interview questions by CFIR construct and CRE-framed evaluation question

**CFIR construct**	**CRE-framed evaluation question**	**Interview/focus group question**
***Outer setting***		
Context	• How do contextual factors (e.g., geographic location, political and social climate, and economic conditions) affect implementation of the programs?	• Describe the community surrounding the program • In your opinion, how does the community environment affect the program?
Patient needs and resources	• To what extent are the programs aware of participant needs, particularly needs that are uniquely experienced by Black, African American, Latino, Hispanic, and African immigrant participants? • To what extent are the programs aware of barriers and facilitators to meeting participants’ needs?	• In your experience, what are the unique needs of the (Black, African American, Latino, Hispanic, and African immigrant) participants in the program? When I say unique needs, I mean needs that participants from these populations may have that are different from the needs of the general population
***Inner setting***		
Leadership engagement	• How does the commitment, involvement, and accountability of leaders and managers impact implementation?	• How much is program leadership committed to integrating culturally tailored strategies into program? Can you provide some examples? Where do they fall short in their commitment?
Partnerships and networks	• How are the programs partnering with other external organizations to implement the programs?	• What role do community-based organizations that represent this population play in implementing the program?
***Individual/team characteristics***		
Other personal attributes	• What traits, skills, and competencies of coaches/educators are important for successful program implementation?	• Complete this sentence: an effective educator in a DSMES program/coach in a lifestyle change program working with your participants needs to be…
***Intervention Characteristics***		
Adaptability	• How are the programs adapted to meet the needs of Black, African American, Latino, Hispanic, and African immigrant participants?	• How has the curriculum been developed to meet the needs of diverse program participants, specifically Black, African American, Hispanic, Latino, and African immigrant participants?

### Step 7: Collect the Data

Primary data were collected during in-person site visits to each of the four programs. For each interview or focus group, we used tailored semi-structured guides developed as part of Step 6. Guides were tailored by site and respondent type to explore unique intervention and program staff characteristics. To ensure participation in the data collection process, the evaluation team reviewed the list of key participants with program directors, worked with local program directors to schedule the interviews and class observations, and discussed site visit logics. Aligned with CRE guidance to gather diverse perspectives from individuals involved in the program (Hood et al., 2015), we interviewed organizational leaders, program implementers, nutritionists, referral partners, clinical staff, and community partners, and we conducted focus groups with lifestyle coaches and health educators. Because of the time required to obtain necessary approvals at the organization and evaluators’ institutions, we were unable to directly engage with program participants; however, the evaluation team observed classes during the site visits. All interviews and focus groups were recorded with permission and, once they were de-identified, were transcribed using a transcription service.

For the quantitative cost data collection, we administered the cost survey developed in Step 6 to gather cost inputs from each site during the site visit. Before administering the survey, we conducted a training with the staff to walk through the tool and answer questions. In addition, we collected secondary data from the sites on program implementation and outcomes.

### Step 8: Analyze the Data

Integrating CFIR into the evaluation and using the framework to inform instrument development yielded efficiencies for the data analysis. Integrating CFIR into the evaluation also enabled us to analyze data in a culturally responsive manner, which included operationalization of the individual domain to explore multiple perspectives as recommended in CRE (Hood et al., 2015). For qualitative analysis, the evaluation team started with a codebook we developed a priori based on the CFIR contextual and the outcome constructs. Evaluators were trained using the codebook, and 20 percent of the transcripts were double coded initially to achieve strong intercoder agreement based on the standard, kappa coefficient ≥ 0.8. The remaining transcripts were coded independently. This deductive coding approach helped us organize the data by construct and allowed us to explore emergent themes and “outlier” perspectives under each construct (Hood et al., 2015; Patton, 1987). For example, interview and focus group protocols included the item, “Complete this sentence: An effective educator in a DSMES program/coach in a lifestyle change program working within your community needs to be …” This item helped address the CRE-framed evaluation question, “What traits, skills, and competencies of lifestyle coaches and diabetes educators are important for successful program implementation with priority populations?,” which maps to the individual/team characteristics CFIR construct. A key theme that emerged from responses to this item was lifestyle change and diabetes educators’ representativeness of the populations being served. In most cases, interview participants emphasized the importance of staffing programs with coaches and educators who represent the priority populations they served (i.e., Black, African American, Latino, Hispanic, and African immigrant populations). However, one interview participant felt strongly that a shared regional culture (e.g., the “Southern food culture”) was also key to building connections between program providers and participants.

For quantitative analysis of data for program reach, dose, and clinical outcomes, the evaluation team analyzed data by race, ethnicity, gender, and age. Although the reach of the evaluated programs was relatively small, ranging from approximately 300 participants in each of the evaluated National DPP programs over a 3-to-4-year period to almost 4900 over a 2-year period in one of the DSMES programs. The other DSMES program enrolled over 2,500 participants over 2 years. Analyzing data by different characteristics is important in CRE to assess whether programs may have differential effects among different population groups (Hood et al., 2015). In our mixed-method approach, relevant quantitative data were reviewed concurrently with qualitative data to bolster interpretation of findings. For example, data on program reach complemented qualitative data about the challenges of recruiting and retaining male participants from underserved populations in the programs. In addition, we analyzed the costs of each program, which is a construct in the CFIR intervention characteristics domain. The data collected covered at least a 12-month period with the aim of obtaining sufficient data for answering the evaluation questions.

The SME panels were also a valuable strategy for engaging health disparities and diabetes experts, including lifestyle change and DSMES program implementors, in the interpretation of findings, particularly regarding divergent perspectives. SMEs also provided additional real-world examples and key insights. For example, regarding lifestyle change coaches’ and diabetes educators’ representativeness of the populations being served, SMEs thought that the ethnicity of the coach relative to the participants is important (i.e., for the purposes of representation and relatability), but coaches whose ethnicity differs from the participants’ can also be successful if they approach the class through a “learning from the participant” perspective.

### Step 9: Disseminate and Use the Results

CRE guidance for dissemination includes engaging stakeholders in a review of evaluation products, as well as sharing evaluation learnings with diverse stakeholders and through different communication formats (Hood et al., 2015). Our evaluation team engaged program directors and SMEs in reviewing an evaluation report that was developed for each of the four sites. The evaluation reports for this work were designed for both internal use by DDT and staff from other CDC chronic disease programs to inform future funding opportunities for state and local health agencies, as well as for internal use by the sites. The sites planned on using these reports to promote their programs within their community and enhance program implementation. Each site report was reviewed by program directors and leaders to ensure that the reports accurately depicted the perspectives of interview and focus group participants and program culture and context.

In addition, evaluation findings and insights gathered during SME panels were compiled in practice-based guides for lifestyle change and DSMES program administrators. The guides included a more simplified and user-friendly version of the integrated CRE-CFIR framework as a roadmap for implementing and evaluating lifestyle change and DSMES programs. Both guides will be made publicly available on the CDC website. Lastly, the methods are summarized in this paper for the broader public health, implementation science, and evaluation communities to demonstrate the value of integrating CRE and CFIR and to promote culturally responsive strategies for implementing and evaluating lifestyle change and DSMES programs.

## Implications for the Field

The integrated CRE and Adapted CFIR approach guided the evaluations of two National DPP lifestyle change and two DSMES programs. Specifically, we considered CFIR constructs in the design, collection, and analysis aspects of the CRE. We used components of the CFIR to help us identify factors that might influence the implementation and effectiveness of strategies aimed at increasing enrollment and retention at the National DPP lifestyle change program and DSMES sites. For example, we considered the contextual and cultural factors included in the community (or outer setting) and within the organizations implementing the program (inner setting); characteristics of the program staff, including educators and lifestyle coaches; and priority populations. As outlined in the Adapted CFIR, we also explored implementation measures, such as the quality and consistency of program delivery, and relevant effectiveness outcomes, such as enrollment and retention in the program and weight loss.

Although this approach was used to evaluate evidence-based diabetes prevention and management interventions, it could be used in other evaluations of multicomponent chronic disease interventions for similar populations. As evidence-based interventions are adapted to best meet the needs of specific populations in real-world settings, it will be important to ensure evaluations account for cultural and contextual factors.

In addition, we used this approach to evaluate organizations offering the lifestyle change and DSMES programs within the community setting, but this approach could also be used to test interventions within health care delivery settings. Cultural and community factors are important to consider even if the intervention is delivered within a health care setting. This integrated evaluation approach was designed to gather practice-based evidence to enhance the implementation of evidence-based programs in real-world settings. Future research to further test this approach among different evidence-based programs and within various settings would be valuable.
